# Supplementary material for: A Likelihood Approach for Real-Time Calibration of Stochastic Compartmental Epidemic Models
Source: PLoS Comput Biol. 2017 Jan 17;13(1):e1005257. doi: 10.1371/journal.pcbi.1005257 (PMC5240920; doi:10.1371/journal.pcbi.1005257)
Supplement: S1 File — (TAR.GZ) [file pcbi.1005257.s014.tar.gz › HSPH_Online-SI-Revision/IPoi09/n1-mild/n1-mild_BENCH_table0.pdf]

1	R0	{1.27677, 7.89375}	{2.14657, 17.9}	{{1.95665, 15.3647}, {2.26161, 19.5128}}	{68.1251, 126.762}	inf	37.	149.864	{102., 202.}	307.696	0.595714	newly	32.	58.661	{45., 79.}	88.5064	0.280051	Reff	0.99205	1.0513	{0.603296, 1.59851}	30.9606	ar	400.	828.119	{787., 863.}	107.03
2	R0	{1.33231, 10.5259}	{2.27671, 16.9865}	{{2.16755, 14.8261}, {2.46477, 19.1373}}	{70.8844, 61.3786}	inf	32.	69.1071	{29., 125.}	122.768	0.306631	newly	17.	25.0714	{10., 46.}	67.0168	0.214007	Reff	0.766078	0.444852	{0.320707, 0.589791}	42.9543	ar	467.	854.232	{814., 898.}	82.9191
3	R0	{1.2273, 6.76944}	{1.46444, 4.12756}	{{1.42949, 3.83841}, {1.49267, 4.47101}}	{19.3223, 39.0265}	inf	26.	17.0313	{1., 46.}	73.1971	0.733959	newly	25.	23.1563	{1., 69.}	88.625	0.713456	Reff	0.996568	0.715913	{0.555014, 0.872915}	28.1621	ar	314.	552.344	{478., 626.}	75.9057
4	R0	{1.41215, 17.5742}	{1.65205, 12.838}	{{1.53473, 10.7075}, {1.76949, 15.6127}}	{16.9882, 27.4981}	inf	25.	63.9091	{32., 101.}	159.273	0.385147	newly	9.	33.0682	{17., 53.}	268.939	0.531606	Reff	1.19468	0.904817	{0.598545, 1.23751}	27.7307	ar	465.	655.773	{553., 736.}	41.0264
5	R0	{1.42222, 6.15886}	{2.44656, 17.2813}	{{2.21411, 14.2406}, {2.66225, 19.9254}}	{72.0238, 180.592}	inf	35.	191.232	{119., 241.}	446.377	0.717477	newly	37.	73.9565	{52., 96.}	102.859	0.299567	Reff	0.924443	0.938864	{0.536876, 1.56807}	33.9939	ar	490.	883.406	{838., 917.}	80.2869
6	R0	{1.24363, 9.95596}	{2.33466, 17.4344}	{{2.03831, 13.7179}, {2.50427, 19.4475}}	{87.7296, 75.1151}	inf	46.	111.971	{29., 190.}	156.65	0.397643	newly	37.	49.8382	{13., 88.}	62.5199	0.274403	Reff	1.06828	1.82112	{1.43407, 2.23458}	70.6651	ar	411.	863.721	{808., 904.}	110.151
7	R0	{1.36478, 5.96056}	{1.57169, 5.32571}	{{1.54465, 4.64686}, {1.59034, 5.30296}}	{15.1609, 14.0519}	inf	43.	25.4483	{6., 66.}	63.5926	0.462793	newly	48.	24.1724	{5., 57.}	61.5661	0.494927	Reff	1.03723	0.668087	{0.483616, 0.820209}	35.5895	ar	484.	626.	{542., 717.}	29.3388
8	R0	{1.36335, 17.6429}	{1.75439, 19.2193}	{{1.73196, 18.9248}, {1.79789, 19.6311}}	{28.6823, 8.9902}	inf	38.	92.8261	{53., 134.}	146.568	0.373445	newly	14.	32.9565	{18., 46.}	136.957	0.350558	Reff	1.17657	1.08831	{0.814019, 1.44509}	19.5015	ar	484.	713.739	{653., 766.}	47.4668
9	R0	{1.26575, 5.28707}	{1.92245, 16.8222}	{{1.85253, 15.8132}, {2.036, 19.2013}}	{51.8825, 218.177}	inf	37.	113.31	{67., 154.}	206.244	0.46802	newly	42.	44.2759	{28., 60.}	21.9212	0.0945877	Reff	0.882228	0.794685	{0.540939, 1.11313}	24.6066	ar	472.	779.276	{738., 826.}	65.1008
10	R0	{1.42942, 9.49905}	{2.2704, 19.2989}	{{2.26107, 19.0355}, {2.30371, 19.5287}}	{58.8338, 103.167}	inf	37.	113.87	{61., 178.}	207.756	0.457547	newly	27.	39.3333	{21., 67.}	57.3806	0.186286	Reff	0.86337	0.589639	{0.377689, 0.929522}	42.5364	ar	468.	854.754	{815., 891.}	82.6397
11	R0	{1.46827, 15.0983}	{1.71677, 19.0642}	{{1.63728, 17.5966}, {1.75814, 19.7105}}	{16.9247, 26.267}	inf	35.	79.88	{42., 107.}	134.286	0.36787	newly	18.	30.48	{14., 41.}	80.4444	0.262584	Reff	1.25831	1.12383	{0.756099, 1.51893}	18.6965	ar	495.	685.52	{633., 730.}	38.4889
12	R0	{1.25751, 13.279}	{1.60733, 17.1828}	{{1.51444, 15.4649}, {1.73196, 19.7105}}	{27.8188, 30.1381}	inf	31.	60.8837	{14., 105.}	119.055	0.363744	newly	18.	25.2326	{5., 42.}	67.8295	0.274623	Reff	1.09906	1.18019	{0.803538, 1.50753}	20.9513	ar	366.	650.14	{572., 712.}	77.6338
13	R0	{1.3425, 9.16361}	{1.91458, 11.7056}	{{1.83592, 11.1369}, {1.98732, 12.4507}}	{42.6133, 27.7401}	inf	30.	47.1	{20., 92.}	76.8889	0.229768	newly	15.	25.2	{9., 50.}	87.7778	0.258839	Reff	0.839063	0.538803	{0.413363, 0.635123}	36.7609	ar	442.	769.533	{723., 808.}	74.1026
14	R0	{1.18936, 6.20583}	{1.9074, 10.7459}	{{1.83592, 9.75343}, {1.98732, 12.1446}}	{60.372, 73.1578}	inf	36.	89.44	{49., 129.}	150.111	0.373088	newly	30.	52.52	{30., 77.}	79.2	0.236463	Reff	0.848014	0.752193	{0.463332, 1.15447}	29.7843	ar	382.	772.5	{713., 818.}	102.225
15	R0	{1.27137, 9.78868}	{1.80513, 10.7566}	{{1.71608, 9.75208}, {1.88607, 11.9453}}	{41.9828, 11.5106}	inf	42.	83.2826	{37., 114.}	105.745	0.301458	newly	20.	50.5435	{23., 72.}	157.5	0.384747	Reff	0.943357	0.8559	{0.577761, 1.22126}	25.7466	ar	496.	723.283	{661., 783.}	45.8231
16	R0	{1.23102, 13.6452}	{1.7924, 13.5399}	{{1.67961, 11.5667}, {1.93288, 15.9062}}	{45.6025, 11.7729}	inf	28.	89.8269	{44., 131.}	226.854	0.496119	newly	7.	45.4808	{22., 66.}	554.121	0.787413	Reff	0.984816	0.960956	{0.636957, 1.49112}	26.9135	ar	445.	725.019	{630., 784.}	62.9257
17	R0	{1.27213, 13.7452}	{1.81003, 14.2889}	{{1.58389, 10.5556}, {2.00043, 17.8178}}	{42.2838, 17.2902}	inf	25.	101.83	{33., 164.}	313.358	0.585243	newly	15.	47.1132	{24., 64.}	218.616	0.485186	Reff	1.11566	1.06278	{0.722799, 1.57296}	22.5762	ar	367.	730.019	{643., 810.}	98.9152
18	R0	{1.25318, 11.2151}	{1.83636, 18.6098}	{{1.79443, 16.8903}, {1.87294, 19.1994}}	{46.5363, 65.935}	inf	34.	99.5833	{52., 137.}	197.696	0.45615	newly	18.	37.6833	{19., 55.}	116.944	0.331697	Reff	0.988759	1.10851	{0.721491, 1.55516}	24.571	ar	416.	748.467	{692., 793.}	79.9199
19	R0	{1.46006, 8.11496}	{2.30359, 14.3848}	{{2.0968, 12.1677}, {2.48143, 16.1449}}	{57.7736, 77.2631}	inf	40.	171.984	{105., 237.}	329.96	0.609885	newly	35.	76.5714	{49., 101.}	120.227	0.330495	Reff	0.995761	0.874971	{0.49934, 1.48373}	34.4798	ar	496.	863.905	{815., 905.}	74.7143
20	R0	{1.14273, 6.71261}	{2.1192, 18.3789}	{{1.95665, 16.2766}, {2.26107, 19.9579}}	{85.4508, 173.797}	inf	37.	149.54	{102., 204.}	306.65	0.588904	newly	33.	57.9365	{41., 75.}	82.7802	0.26783	Reff	0.861618	1.1025	{0.648313, 1.55189}	40.2702	ar	362.	826.746	{772., 866.}	128.383
21	R0	{1.42432, 8.23905}	{1.58839, 7.23757}	{{1.58886, 7.29751}, {1.58886, 7.29751}}	{11.5189, 12.1552}	inf	21.	17.3	{1., 38.}	71.9048	0.566377	newly	20.	14.4	{1., 39.}	70.3333	0.583932	Reff	1.02409	0.630726	{0.530679, 0.727698}	38.4108	ar	405.	630.267	{543., 685.}	55.6214
22	R0	{1.29386, 17.863}	{1.77582, 15.2019}	{{1.5767, 11.6931}, {1.98971, 19.2013}}	{37.2502, 18.1355}	inf	30.	84.	{30., 146.}	193.725	0.488111	newly	13.	39.1176	{13., 60.}	212.368	0.506906	Reff	1.13601	1.14475	{0.758143, 1.62175}	27.4782	ar	488.	698.216	{624., 810.}	46.7012
23	R0	{1.39274, 13.0167}	{1.70855, 11.5881}	{{1.63746, 10.1332}, {1.79436, 13.7237}}	{22.6755, 13.7011}	inf	50.	26.6939	{1., 81.}	66.3673	0.691493	newly	23.	13.8776	{1., 39.}	67.3469	0.655492	Reff	0.997202	0.605874	{0.443207, 0.854407}	39.2426	ar	475.	688.449	{624., 767.}	44.9366
24	R0	{1.32178, 15.8991}	{1.97831, 18.6839}	{{1.85253, 16.0085}, {1.99771, 19.6444}}	{49.6702, 17.9827}	inf	70.	118.678	{53., 168.}	80.7748	0.260837	newly	28.	45.8644	{20., 65.}	74.6973	0.244628	Reff	1.00059	1.10078	{0.681219, 1.7036}	31.0889	ar	471.	784.	{746., 831.}	66.4544
25	R0	{1.37568, 17.8427}	{1.60077, 12.9271}	{{1.53432, 11.4771}, {1.67042, 14.7029}}	{16.3617, 27.5498}	inf	28.	54.7556	{19., 88.}	106.19	0.299745	newly	9.	28.4222	{10., 47.}	220.247	0.458569	Reff	1.11843	0.817388	{0.58102, 1.1605}	30.1664	ar	398.	645.711	{573., 701.}	62.239
26	R0	{1.20993, 6.4656}	{1.91935, 14.9537}	{{1.78373, 11.799}, {2.05052, 16.7932}}	{58.6329, 131.281}	inf	26.	107.702	{57., 162.}	322.47	0.628781	newly	24.	47.3684	{27., 67.}	107.018	0.340833	Reff	0.908657	0.931288	{0.575754, 1.50282}	27.3305	ar	345.	752.614	{667., 826.}	121.281
27	R0	{1.20548, 13.8003}	{1.86947, 19.0568}	{{1.8189, 18.7084}, {1.91377, 19.3194}}	{55.0811, 38.0899}	inf	33.	116.273	{75., 158.}	254.766	0.536129	newly	20.	42.9818	{25., 57.}	117.818	0.326105	Reff	0.947507	0.998367	{0.709844, 1.35975}	23.5608	ar	350.	752.782	{710., 795.}	115.081
28	R0	{1.3176, 14.6594}	{1.7948, 17.4669}	{{1.64552, 14.5038}, {1.93087, 19.3194}}	{36.2177, 20.9469}	inf	28.	76.6071	{19., 137.}	183.673	0.416419	newly	16.	30.6071	{9., 52.}	107.366	0.314294	Reff	1.18979	1.41776	{1.15403, 1.67551}	22.0771	ar	362.	738.75	{661., 804.}	104.075
29	R0	{1.28202, 6.23047}	{2.19452, 19.0063}	{{2.0818, 17.5151}, {2.26107, 19.9579}}	{71.177, 205.053}	inf	21.	148.868	{96., 199.}	609.454	0.829174	newly	22.	51.9853	{30., 73.}	137.901	0.359286	Reff	0.937157	0.863147	{0.531351, 1.27524}	31.3168	ar	391.	837.074	{802., 874.}	114.085
30	R0	{1.28177, 8.83252}	{1.93083, 11.3768}	{{1.83592, 9.90957}, {2.08003, 13.1443}}	{50.6381, 28.819}	inf	41.	105.827	{59., 152.}	160.46	0.395406	newly	27.	59.6154	{34., 87.}	123.789	0.331249	Reff	0.9562	0.774833	{0.520748, 1.12442}	29.7103	ar	427.	775.5	{730., 818.}	81.6159
31	R0	{1.35886, 5.97708}	{2.58644, 19.3384}	{{2.46976, 17.5902}, {2.66225, 19.9254}}	{90.3386, 223.542}	inf	33.	197.028	{140., 257.}	497.306	0.758652	newly	34.	67.8611	{47., 88.}	102.206	0.297253	Reff	0.841134	0.929682	{0.470584, 1.60739}	42.2278	ar	491.	899.403	{865., 926.}	83.1778
32	R0	{1.27601, 17.9804}	{1.77686, 18.5403}	{{1.66003, 15.8482}, {1.84468, 19.9256}}	{39.251, 8.57172}	inf	45.	87.375	{32., 128.}	106.111	0.303753	newly	24.	34.3542	{15., 52.}	59.9826	0.217998	Reff	1.09737	1.15345	{0.763827, 1.56956}	21.1907	ar	445.	713.875	{647., 775.}	60.4213
33	R0	{1.3368, 11.3235}	{2.14914, 18.5276}	{{1.97176, 16.2766}, {2.26107, 19.9579}}	{60.7677, 63.621}	inf	59.	155.918	{112., 201.}	169.658	0.431459	newly	32.	59.8197	{42., 80.}	92.8791	0.288027	Reff	0.974527	1.0919	{0.682167, 1.49163}	30.4917	ar	417.	829.098	{787., 866.}	98.8245
34	R0	{1.21021, 11.3635}	{1.45107, 8.11582}	{{1.42446, 7.81552}, {1.54207, 9.08517}}	{19.9019, 28.5799}	inf	17.	29.0345	{4., 63.}	100.406	0.337708	newly	9.	22.	{4., 45.}	167.433	0.42955	Reff	1.00689	0.834523	{0.588538, 1.22803}	25.4913	ar	327.	569.241	{480., 655.}	74.0799
35	R0	{1.53382, 10.8866}	{2.21416, 18.671}	{{2.12978, 17.6755}, {2.27287, 19.5128}}	{44.3558, 71.5041}	inf	51.	162.774	{114., 208.}	221.316	0.498924	newly	30.	62.8387	{42., 82.}	113.226	0.325194	Reff	1.1565	1.1422	{0.716185, 1.55146}	24.2881	ar	476.	841.339	{815., 871.}	76.7518
36	R0	{1.20423, 6.83844}	{2.60291, 18.4307}	{{2.315, 15.4658}, {2.79269, 19.984}}	{116.147, 169.516}	inf	51.	221.408	{159., 265.}	336.012	0.631971	newly	46.	82.1127	{65., 97.}	81.9963	0.262947	Reff	0.814059	1.01993	{0.538828, 1.53233}	39.7228	ar	416.	901.38	{865., 931.}	116.678
37	R0	{1.43629, 10.1548}	{1.8315, 15.0605}	{{1.67375, 12.378}, {1.8951, 16.0085}}	{27.5158, 48.3089}	inf	42.	85.2353	{42., 125.}	110.504	0.31249	newly	33.	37.3725	{20., 55.}	37.8491	0.181433	Reff	1.10594	0.810671	{0.582465, 1.24049}	34.5982	ar	367.	738.667	{672., 790.}	101.272
38	R0	{1.37504, 8.93428}	{2.3495, 15.5045}	{{2.1245, 12.644}, {2.66225, 19.6025}}	{70.8676, 73.5391}	inf	75.	165.676	{99., 232.}	122.471	0.332324	newly	60.	71.4412	{43., 95.}	30.6373	0.122072	Reff	0.897901	0.805549	{0.476034, 1.27376}	32.0522	ar	483.	860.632	{806., 920.}	78.1848
39	R0	{1.51637, 9.36742}	{1.95423, 11.5312}	{{1.87584, 10.2217}, {2.04669, 12.9719}}	{28.8758, 23.0989}	inf	30.	76.5	{29., 132.}	162.564	0.385046	newly	16.	42.0769	{13., 74.}	172.115	0.399116	Reff	1.019	0.677027	{0.430648, 0.999715}	40.1806	ar	441.	779.577	{742., 825.}	76.7748
40	R0	{1.23658, 9.82869}	{1.99334, 11.1991}	{{1.86868, 9.28761}, {2.20345, 14.058}}	{61.1976, 15.9151}	inf	71.	110.571	{45., 165.}	67.9074	0.219035	newly	51.	61.4464	{31., 92.}	40.2311	0.159419	Reff	0.868079	0.802914	{0.511619, 1.23528}	29.2818	ar	414.	792.554	{722., 847.}	91.4381
41	R0	{1.24247, 5.4242}	{1.87506, 9.13254}	{{1.75445, 7.55154}, {1.96658, 10.2409}}	{50.9142, 68.3666}	inf	30.	98.9184	{45., 153.}	233.673	0.500561	newly	38.	74.9796	{35., 115.}	104.834	0.304543	Reff	0.956702	0.953121	{0.594258, 1.37909}	27.7106	ar	345.	750.714	{668., 800.}	117.598
42	R0	{1.28208, 4.40223}	{2.58513, 18.2374}	{{2.315, 15.4658}, {2.79269, 19.984}}	{101.636, 314.277}	inf	25.	208.394	{132., 267.}	733.577	0.893799	newly	44.	83.2394	{60., 105.}	94.0461	0.288624	Reff	0.951303	1.34337	{0.851347, 1.89466}	47.3015	ar	339.	898.085	{867., 924.}	164.922
43	R0	{1.56773, 6.50474}	{2.27969, 9.2827}	{{2.12684, 8.23415}, {2.47628, 10.2848}}	{45.4133, 42.7067}	inf	43.	132.779	{75., 192.}	210.431	0.467647	newly	49.	87.3676	{45., 135.}	84.5438	0.248836	Reff	0.981399	0.653506	{0.39497, 1.07393}	42.1825	ar	498.	857.338	{816., 892.}	72.1563
44	R0	{1.10489, 6.01941}	{2.02429, 17.655}	{{1.92096, 15.3647}, {2.12588, 19.5406}}	{83.2123, 193.3}	inf	33.	127.15	{81., 183.}	285.303	0.565303	newly	35.	46.0833	{29., 64.}	39.381	0.139866	Reff	0.79994	0.809539	{0.584617, 1.13094}	19.9815	ar	346.	796.883	{736., 841.}	130.313
45	R0	{1.25888, 14.1926}	{2.04381, 18.2743}	{{1.93288, 16.0085}, {2.1968, 19.9579}}	{62.3517, 28.7592}	inf	65.	117.207	{65., 163.}	83.7666	0.249387	newly	24.	42.0172	{24., 61.}	80.1006	0.247726	Reff	0.815754	0.732696	{0.482575, 1.06247}	28.1195	ar	471.	804.31	{757., 848.}	70.7665
46	R0	{1.45292, 16.616}	{1.93528, 14.6622}	{{1.72065, 11.7288}, {2.21213, 18.788}}	{33.1995, 17.2001}	inf	45.	121.983	{65., 180.}	173.296	0.412918	newly	23.	56.8	{36., 78.}	148.841	0.3827	Reff	1.20738	1.08287	{0.628285, 1.4832}	23.8899	ar	494.	771.267	{685., 855.}	56.1269
47	R0	{1.22528, 16.6033}	{1.6103, 14.8997}	{{1.44158, 10.9674}, {1.80508, 19.4054}}	{31.423, 17.1776}	inf	28.	57.3636	{13., 114.}	132.955	0.378076	newly	12.	27.9091	{8., 52.}	150.379	0.401469	Reff	1.1101	1.33579	{1.05061, 1.6159}	22.9329	ar	368.	628.5	{515., 738.}	78.6314
48	R0	{1.1669, 6.92715}	{1.91598, 17.3854}	{{1.83921, 15.8132}, {1.99771, 19.2013}}	{64.1938, 150.975}	inf	37.	113.633	{64., 154.}	207.115	0.467297	newly	38.	43.551	{25., 58.}	30.1826	0.122797	Reff	0.839001	0.835881	{0.592777, 1.16456}	23.0286	ar	416.	771.633	{730., 818.}	85.4886
49	R0	{1.32749, 16.0887}	{1.75075, 17.4005}	{{1.59546, 13.3174}, {1.84468, 19.688}}	{31.8841, 14.0223}	inf	27.	94.8667	{43., 141.}	261.399	0.565309	newly	11.	36.6667	{20., 52.}	241.01	0.550257	Reff	1.17085	1.15259	{0.796221, 1.54215}	18.7181	ar	350.	700.733	{664., 780.}	102.348
50	R0	{1.29636, 15.2609}	{2.02591, 17.7021}	{{1.92096, 15.3647}, {2.12978, 19.5406}}	{56.2767, 16.1975}	inf	42.	132.228	{68., 191.}	214.829	0.475872	newly	19.	47.807	{25., 70.}	151.616	0.375837	Reff	0.917823	0.789508	{0.549395, 1.18292}	25.2782	ar	470.	805.667	{764., 846.}	71.4184
